# Oral Health-Related Quality of Life Changes in Patients with Dentofacial Deformities Class II and III after Orthognathic Surgery: A Systematic Review and Meta-Analysis

**DOI:** 10.3390/ijerph19041940

**Published:** 2022-02-09

**Authors:** Valentina Duarte, Carlos Zaror, Julio Villanueva, Matías Andreo, Matías Dallaserra, Josefina Salazar, Àngels Pont, Montse Ferrer

**Affiliations:** 1 Faculty of Sciences, Pontificia Universidad Católica de Valparaíso, Valparaíso 2340000, Chile; 2 Oral & Maxillofacial Surgery, Hospital Carlos Van Buren, Valparaíso 2340000, Chile; matias.andreo@gmail.com; 3 Department of Paediatrics, Obstetrics and Gynaecology and Preventive Medicine Universitat Autònoma de Barcelona, 08193 Barcelona, Spain; 4 Department of Paediatric Dentistry and Orthodontic, Faculty of Dentistry, Universidad de La Frontera, Temuco 4780000, Chile; 5 Center for Research in Epidemiology, Economics and Oral Public Health (CIEESPO), Faculty of Dentistry, Universidad de La Frontera, Temuco 4780000, Chile; 6 Department of Oral & Maxillofacial Surgery, Faculty of Dentistry, Universidad de Chile, Santiago 8380000, Chile; javm@uchile.cl (J.V.); matias.dallaserra@gmail.com (M.D.); 7 Cochrane Associated Center, Faculty of Dentistry, University of Chile, Santiago 8380000, Chile; 8 Department of Oral & Maxillofacial Surgery, Hospital Clínico San Borja-Arriarán, Santiago 8320000, Chile; 9 Iberoamerican Cochrane Centre, 08025 Barcelona, Spain; josefinasalazarn@gmail.com; 10 Health Services Research Group IMIM, Hospital del Mar Medical Research Institute, 08003 Barcelona, Spain; apont@imim.es (À.P.); mferrer@imim.es (M.F.); 11 CIBER en Epidemiología y Salud Pública, CIBERESP, 28029 Madrid, Spain; 12 Department of Experimental and Health Sciences, Pompeu Fabra University, 08003 Barcelona, Spain

**Keywords:** oral health-related quality of life, orthognathic surgery, dentofacial deformity

## Abstract

Our aim was to assess the impact of combined orthodontic–surgical treatment on patients’ oral health-related quality of life (OHRQoL) according to type of dentofacial deformities, by synthesizing the available evidence. Methods: Search was conducted in the PubMed, Embase/MEDLINE, Scopus, and Cochrane databases. The eligibility criteria were studies that measured OHRQoL before–after orthognathic surgery, with results disaggregated by Class II and III. Two researchers independently performed the selection process, data extraction, and methodological quality assessment. Meta-analysis of the standard mean differences (SMD) was performed using random effect models. Results: The search identified 1047 references. Thirteen studies met the inclusion criteria, and four were included in the meta-analysis. The SMD of OHRQL global score showed large improvement 4–7 months after surgery in Class II and III patients (2.09, 95% CI 0.68 to 3.49 and 1.96, 95% CI 1.22 to 2.70, respectively). The sensitivity analyses, excluding studies with weak methodological quality, showed that Class III patients’ improvement in functional limitation was significantly higher than in Class II patients (SMD 0.57, 95% CI 0.12–1.02). Conclusions: There is not enough evidence to support differences between Class II and III patients in the OHRQoL impact after orthognathic surgery, but findings suggest lower improvement of some domains in Class II patients.

## 1. Introduction

Dentofacial deformities refer to significant deviations from normal proportions of the maxillo-mandibular complex, being one of the oral health problems most perceived by the population. This condition affects the quality of social relationships, self-esteem [1,2], and oral health-related quality of life (OHRQoL) [2,3], which has been defined as a “multidimensional construct that includes a subjective evaluation of the individual’s oral health, functional well-being, emotional well-being, expectations and satisfaction with care, and sense of self” [3].

The combination of orthodontic and orthognathic surgery is the most established treatment to correct Class II and Class III dentofacial deformities [4,5,6,7]. The main objective of orthognathic surgery is to correct the facial skeleton, to facilitate malocclusion orthodontic therapy. Interest from traditional clinical outcomes of orthognathic surgery (aesthetic, functional, planning, surgical technique, and complications) [8,9,10,11] has moved to OHRQoL since the beginning of the 21st century to incorporate the patients’ perspective [12,13,14]. Achieving a better quality of life in patients with dentofacial deformities is one of the objectives of the treatment, based mainly on the need to improve aspects related to aesthetic, functional, and psychosocial factors [13,15,16].

The first systematic review [17] about quality of life assessment in patients with dentofacial deformities undergoing orthognathic surgery, published in 2013, described the different motivations and perceptions of patients towards surgical treatment, the methods and instruments used to measure quality of life and psychosocial aspects, but not the changes between before and after surgery. Subsequently, five systematic reviews, two with narrative synthesis [18,19], and the other three with quantitative synthesis through meta-analysis [20,21,22], have focused on this outcome, showing OHRQoL improvement. None of these systematic reviews stratified by type of dentofacial deformity, despite consistently reported differences between Class II and Class III patients [15,23,24,25].

The aim of this study was to assess the impact of combined orthodontic–surgical treatment on OHRQoL in patients with dentofacial deformities of Class II and Class III by synthesizing the available evidence through a systematic review with meta-analysis.

## 2. Material and Methods

### 2.1. Protocol and Registration

A systematic review was conducted in accordance with the Cochrane Handbook for the Systematic Review of Interventions [26] and reported according to the guidelines of the Preferred Reporting Items for Systematic Reviews and Meta-Analyses (PRISMA 2020 statement) [27] (Supplementary Table S2). The study was registered at the International Prospective Register of Systematic Reviews (PROSPERO) from the National Institute for Health Research database (www.crd.york.ac.uk/prospero) (accessed on 17 September 2021) with registration number CRD42019116092.

Following the PICO (patient, intervention, comparison, outcome) framework, our research question was: In patients with dentofacial deformities of Class II and Class III (P), does the combined orthodontic–surgical treatment (I) have an impact (C) on their oral health-related quality of life (O)? Impact here refers to the comparison between before and after treatment, with or without control group.

### 2.2. Elegibility Criteria

Inclusion criteria: randomized and nonrandomized clinical trials with or without control group, in patients with dentofacial deformities submitted to combined orthodontic–surgical treatment, that measured OHRQoL before and after surgery; including patients over 15 years old; using validated OHRQoL instruments; and presenting results disaggregated by dentofacial deformity of Class II and Class III. Exclusion criteria: patients undergoing a surgery-first approach or sleep apnea surgical treatment; studies related to patients with congenital abnormalities, such as craniofacial syndrome or cleft lip and palate, and sequels due to maxillofacial trauma; using only generic instruments to assess HRQoL, such as SF36 or EQ5D, not those specific for oral health; assessing psychometric properties; case reports or cases series or studies that were not primary; and not published in English, Spanish, German, or Portuguese.

### 2.3. Information Sources and Search Strategy

Searches for eligible articles were undertaken in four databases—PubMed, Embase/MEDLINE, Scopus, and the Cochrane Central Register of Controlled Trials (CENTRAL)—from their inception to October 2021. The following terms were used in the search: “dentofacial deformities”, “orthognathic surgery”, and “quality of life”. No limits of date or languages were added to the searches since the first orthognathic surgery was described in 1849. The details of the search strategy used in each database are listed in the supplementary data (Supplementary Table S1). Gray literature was explored by reviewing reference lists of selected primary studies and other published systematic reviews to identify studies.

### 2.4. Selections Process

The systematic review followed three stages using COVIDENCE online software (Veritas Health Innovation, Melbourne, Australia) (www.covidence.org) (accessed on 21 November 2021): (1) title- and abstract-screening; (2) full-text review with data extraction; and (3) review of references listed in articles. Title- and abstract-screening were performed independently by two reviewers of the study team (V.D. and M.D.), based on the inclusion and exclusion criteria previously established; disagreement was resolved by consensus or by a third reviewer (C.Z.), who acted as an arbitrator. Subsequently, all the selected articles were independently full-text reviewed by two reviewers (V.D. and M.A.).

### 2.5. Data Collection Process

Data extraction of the studies was conducted independently by two investigators (V.D. and M.A.) using a standardized, predefined collection form that was piloted prior to its use. In order to obtain data which were not provided in the articles of interest, the authors of these studies were contacted.

### 2.6. Data Items

The information extracted from the included studies was publication data, study design, country in which the study was conducted, sample size, patient characteristics, type of dentofacial deformity, OHRQoL instrument used, follow-up data collection times, and results obtained from each group evaluated (mean and standard deviation of global and domain scores). We did not consider missing data as a reason to exclude any of the trials from the review. We did not carry out data imputation, as we assumed all missing data to be at random.

### 2.7. Study of Methodological Quality

The methodological quality was assessed with the Effective Public Health Practice Project (EPHPP) quality assessment tool [28,29] for quantitative studies, which has six components: (a) selection bias, (b) study design, (c) confounders, (d) blinding, (e) data collection methods, and (f) withdrawals/dropouts. Each component was classified as “strong”, “moderate”, or “weak”, and a global rating was obtained according to the number of components rated as weak (0, 1, or >1) [28]. Studies with weak methodological quality had a higher risk of bias. Two researchers (J.S. and M.D.) performed the risk of bias assessment independently; any disagreements were resolved by a third researcher (C.Z.).

### 2.8. Effect Measures and Synthesis Methods

A narrative description was carried out using the characteristics and main results of all studies that fulfilled inclusion criteria. Since most studies assessed OHRQoL immediately prior to surgery (with presurgical orthodontic treatment) and 4–7 months after surgery, change between these time assessments was selected as the main outcome of interest for quantitative synthesis. When mean and SD of change were not reported, mean and SD at each evaluation were collected to calculate the standardized mean difference (SMD) between both evaluations, and SD was estimated with the formula [26]:

(1) $$\mathrm{SD}\mathrm{change}=\sqrt{\mathrm{SD}2\mathrm{baseline}+\mathrm{SD}2\mathrm{final}-\left(2\times \mathrm{Corr}\times \mathrm{SDbaseline}\times \mathrm{SDfinal}\right)}$$

The magnitude of SMD was considered small for 0.2, moderate for 0.5, and large for 0.8 [30].

Forest plots were constructed showing the summary and 95% CI estimated in the meta-analyses, together with results from individual studies. We used a random effect model (the DerSimonian–Laird method), as we expected variation in effects due to differences in study populations, questionnaires, and methods. First, we estimated the SMD of global scores separately for Class II and Class III patients and performed subgroup analysis according to the OHRQoL instrument, as a potential source of heterogeneity. Second, we estimated the SMD of dimension scores, performing subgroup analysis according to Class II and Class III patients, to examine differences between them. Third, we also estimated the difference between SMD in Class II and Class III. Finally, sensitivity analyses were carried out by excluding studies with weak methodological quality. Heterogeneity among studies was evaluated using the I^2^ statistic, categorized as follows: <30% not important; 30–50% moderate; 50–75% substantial; and 75–100% considerable [26]. Funnel plots were planned to explore possible publication bias.

The software used was Review Manager 5.4 (Cochrane IMS, Copenhagen, Denmark).

### 2.9. Certainty Assessment

The Grading of Recommendations Assessment, Development, and Evaluation (GRADE) system was used to assess the overall quality of evidence per comparison and outcome [31]. We constructed a “Summary of Findings” table using GRADEpro GDT software (http://gdt.guidelinedevelopment.org) (accessed on 18 November 2021). The GRADE approach appraises the quality of a body of evidence based on the extent to which one can be confident that an estimate of effect or association reflects the outcome being assessed. We assessed the quality of the body of evidence with reference to the overall risk of bias of the included studies, directness of the evidence, inconsistency of the results, precision of the estimates, risk of publication bias, and magnitude of the effect. The quality of the evidence can be downgraded by one or two levels for each of these factors, reducing the confidence in the estimate of the effect. There are three factors that can increase the quality of evidence: large magnitude of an effect, dose–response gradient, and effect of plausible residual confounding. We categorized the quality of the body of evidence for each of the primary outcomes as high, moderate, low, or very low.

## 3. Results

### 3.1. Study Selection

**Figure 1:**
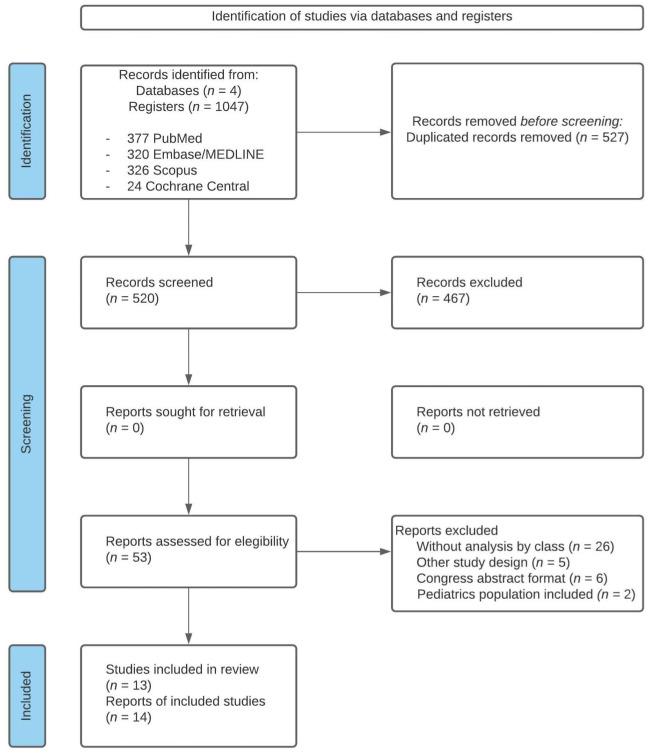
Flow chart of systematic literature review.

The search identified 1047 references (Figure 1); after the removal of duplicates, 520 were screened for title and abstract, and the 53 articles selected according to the eligibility criteria were fully read.

Among the 53 full-text articles reviewed, 39 were excluded for the following reasons: 26 studies had no analysis by type of deformity, 5 were not before–after studies, 6 were congress abstracts, and 2 included a pediatric population (participants under 15 years of age). Finally, 13 studies (14 articles) were included in our qualitative synthesis, and 4 in our quantitative synthesis (meta-analysis).

### 3.2. Study Characteristics

Details of the included studies are summarized in Table 1. Among the 13 prospective before–after studies included, only 3 had a control group composed of: female students at the university who had a normal occlusion (*n* = 14) [32], volunteers aged 19–20 years old attending a nonmedical, specialty university and with no jaw deformities (*n* = 96) [24]; and healthy individuals, mainly patients’ relatives, classmates, or colleagues (*n* = 24) [33]. The final sample size of patients with dentofacial deformities ranged from 14 to 85 subjects, and the mean of age ranged from 21.3 to 31 years. Seven studies used the Oral Health Impact Profile (OHIP-14) [13,15,25,34,35,36,37] to measure OHRQoL, one used the modified Japanese version of OHIP-49 (OHIPJ54) [32], two used the Orthognathic Quality of Life Questionnaire (OQLQ) [23,38], and three studies used both OHIP-14 and OQLQ [24,33,39]. Pre-surgical OHRQoL assessment was carried out during the orthodontic treatment and the last follow-up, around 6 months after surgery in most studies.

The reasons why certain studies could not be included in the meta-analysis were: pre-surgery assessment carried out prior to orthodontic appliance installation [13,34], or after setting orthodontic appliances but at an undetermined time or far from surgery [33,38]; no assessment at 4–7 months after surgery [39]; mean, SD or number of participants by class not reported [32,35,36]; and results provided only at item level [37].

### 3.3. Risk of Bias within Studies

**Figure 2:**
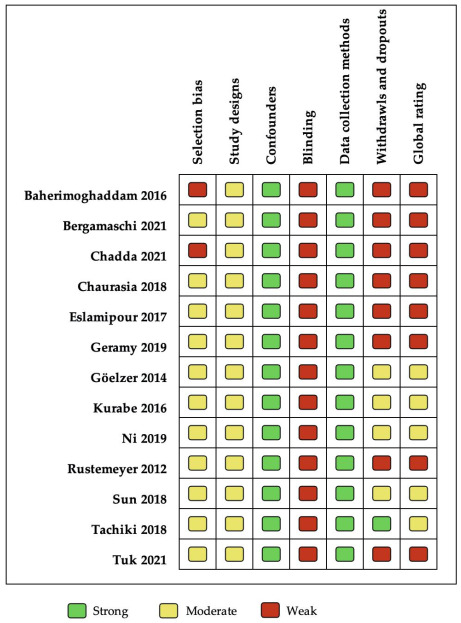
Methodological quality assessed by EPHPP.

Figure 2 shows that five studies were rated as having a moderate methodological quality [24,25,32,33,38], and eight were qualified as being of weak quality, according to the global rating [13,15,23,34,35,36,37,39]. “Data collection methods” was the best evaluated domain, with all studies showing strong quality because they had used a validated OHRQoL instrument. All the studies included were qualified moderate in “study design” because they were before–after studies. “Blinding” was qualified as weak, due to most studies reporting that study participants were not blinded to the research questions. The “Confounders” component was qualified as strong since before–after studies are characterized by the fact that each individual compares with themself, that is, they are their own control. The “selection bias” was moderate because of the limited representativity of the sample. Finally, “withdrawals/dropouts” was the most variable item, with five studies classified as weak mainly because the data was not reported. No study was qualified as having strong methodological quality in the “global rating”, mainly due to nonblinding and the withdrawals/dropouts reported.

### 3.4. Results of Individual Studies

All the included studies showed an improvement in the OHRQoL, regardless of the questionnaire used. The single study providing information on the OQLQ domains [23] reported a significant improvement in all of them (social aspects, dentofacial aesthetics, oral function, and awareness of dentofacial deformity). The facial aesthetics domains of the OQLQ [23,24,39] and the psychological domains of the OHIP-14 presented the greatest improvement at 6 months after surgery [15,24,25,34].

Two studies reported no difference between Class II and III patients [23,35], while another study [32] reported significant differences in the global score and all OHIP-14 domains except functional limitation. No statistically significative change in Class II patients was observed in some studies for functional limitation [25,32,36] and physical disability domains [36], and Sun et al. [24] reported no significant improvement in any domain. Findings from two studies with Class III patients (Tachiki et al. [38] and Ni et al. [33]) showed significant improvement in global and all domain scores, except for awareness [33,38] and social aspects [33].

Baherimoghaddam et al. [15] reported a significative worsening during the pre-surgical stage in OHIP-14 overall score and functional limitation, physical disability, and psychological disability domains in Class II patients. A significant worsening in the domain of functional limitation and physical disability was also observed in Class III patients. However, the global score and all domains in both classes showed a significant improvement in the OHRQoL from before the installation of pre-surgical orthodontic appliances to 6 months after surgery.

### 3.5. Synthesis of Results

Of the four studies that provided data before surgery and 4–7 months after surgery to be included in the meta-analysis, two used the OHIP-14 [15,25], one the OQLQ [23], and one study used both instruments [24].

#### 3.5.1. OHRQL Changes in Class II and Class III Patients Measured with Global Scores of OQLQ and OHIP-14

**Figure 3:**
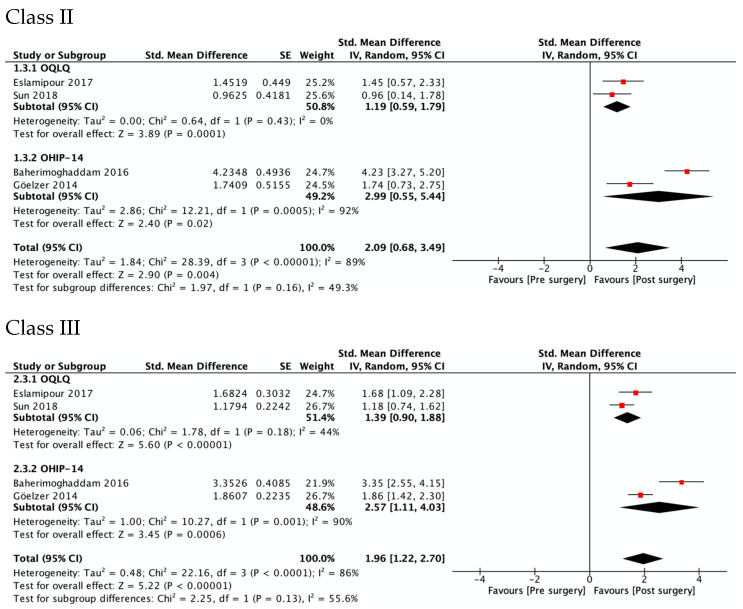
Meta-analysis of the change from pre-surgery to 4–7 months after surgery on the OHRQoL global scores by type of dentofacial deformity.

Figure 3 shows OHRQoL improvement at 4–7 months after surgery in Class II (SMD 2.09, 95% CI 0.68 to 3.49; *I*^2^ = 89%; very low quality of evidence) and Class III patients (SMD 1.96, 95% CI 1.22 to 2.70; *I*^2^ = 86%, low quality of evidence). No differences were observed between the estimators from the two questionnaires, OQLQ and OHIP-14 (*p* value 0.16 in Class II and 0.13 in Class III patients). Regarding the study of Sun et al., OQLQ data was selected for these forest plots because it was designed specifically to measure the impact of orthognathic surgery, while the OHIP-14 is an OHRQL generic instrument.

#### 3.5.2. OHRQL Changes in Class II and Class III Patients in OHIP-14 Domains

**Figure 4:**
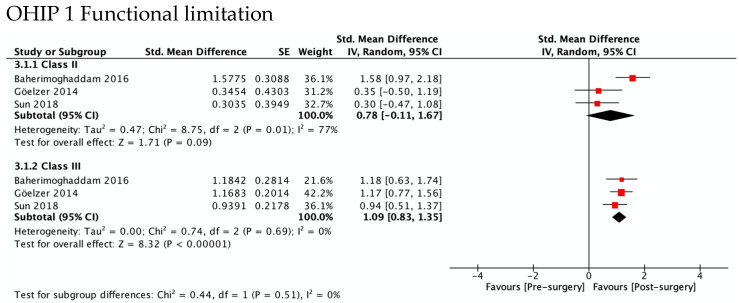

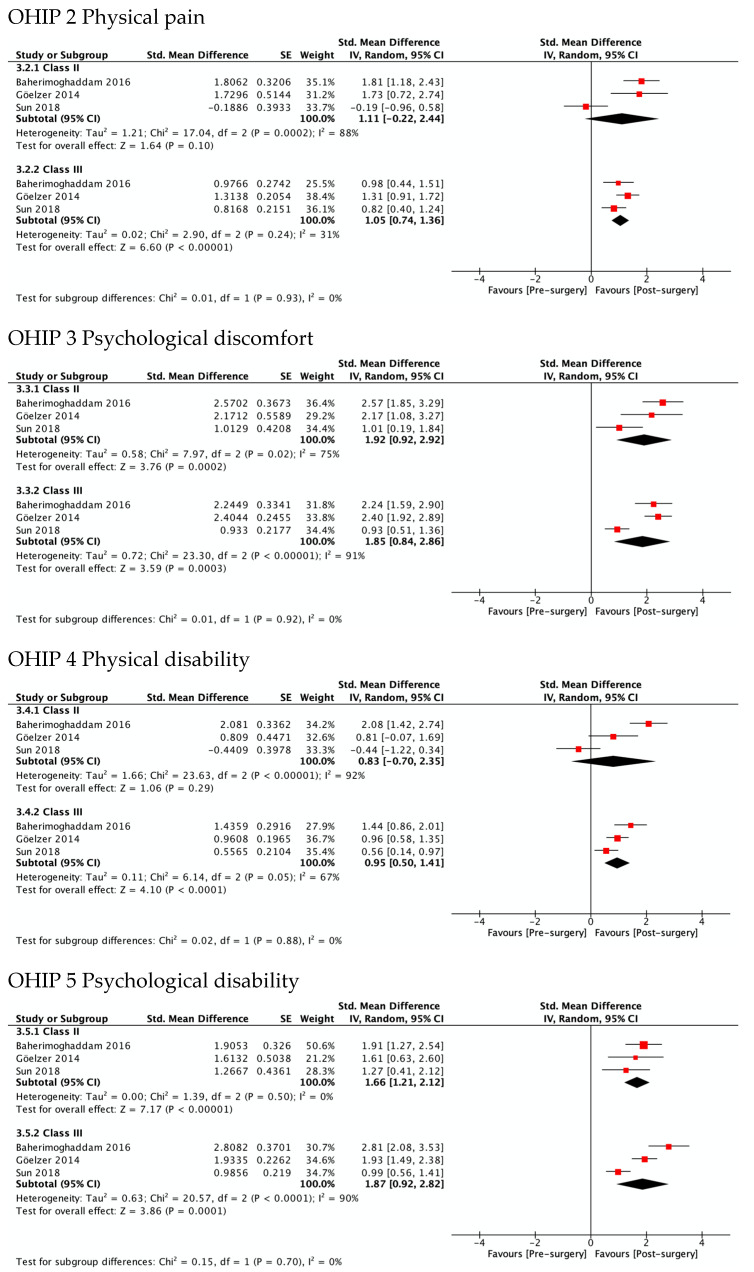

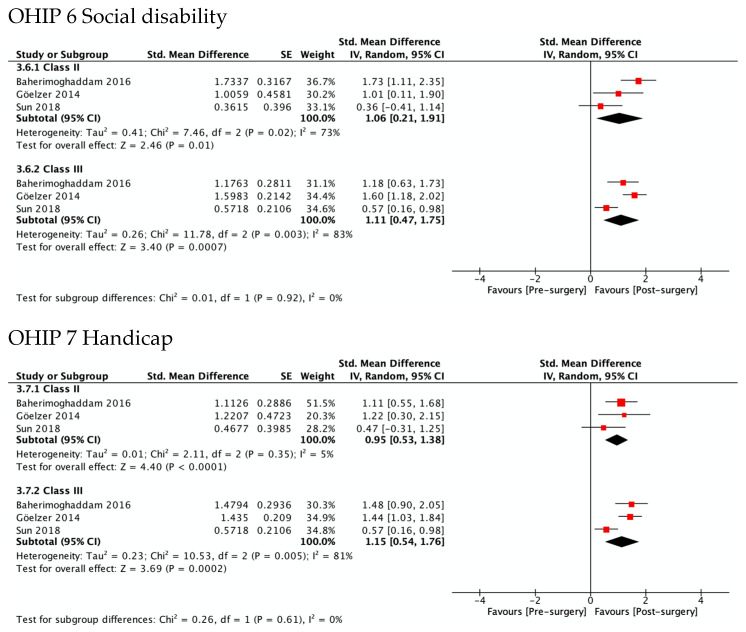
Meta-analysis of the standard mean differences of the OHIP-14 domains’ scores pre-surgery and post-surgery 4–7 months follow-up by type of deformity Class II and III.

**Figure 5:**
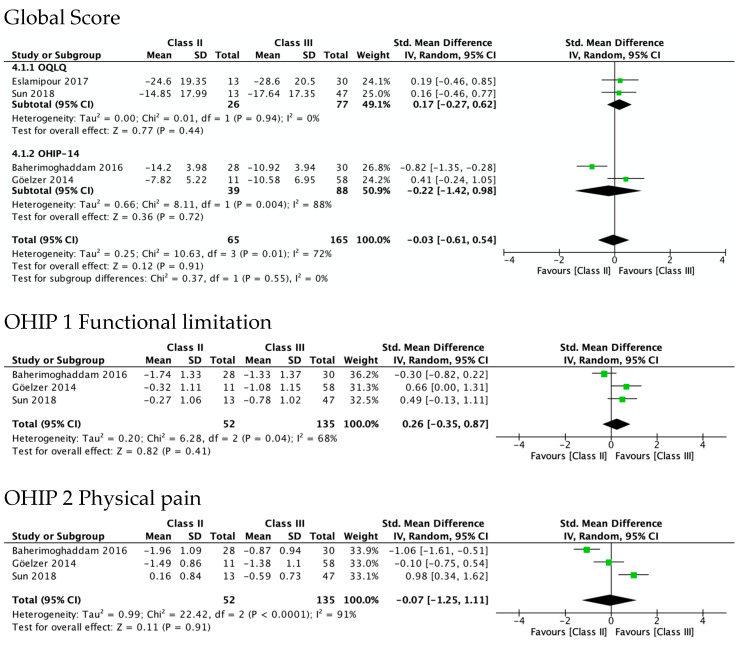

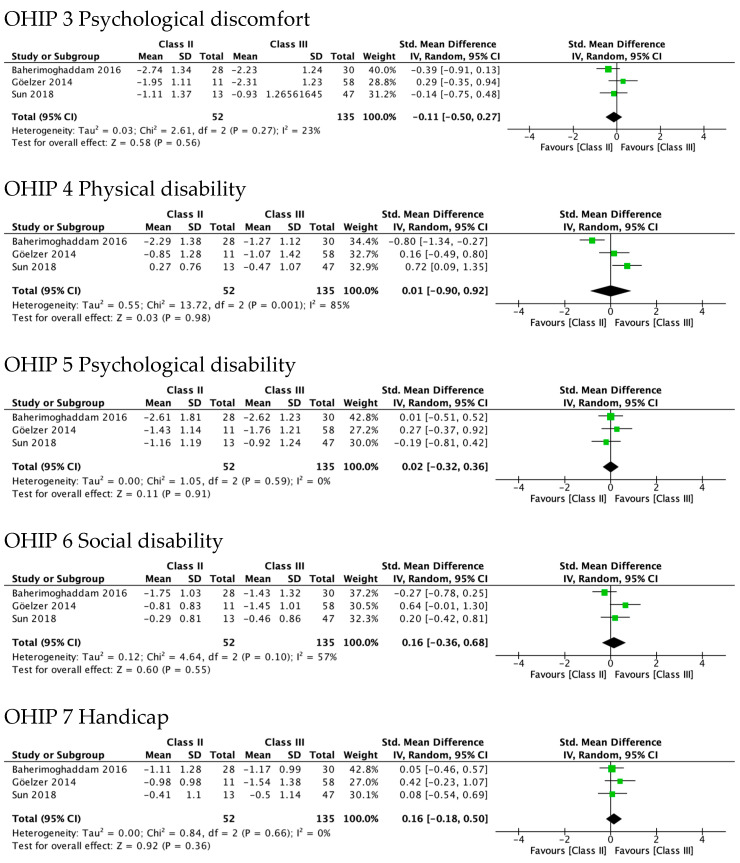
Meta-analysis of the differences between Class II and Class III standard mean differences (from pre-surgery to 4–7 months after surgery) in OHRQoL global scores and OHIP-14 domains’ scores.

Figure 4 shows the greatest improvement for both Class II and Class III in psychological discomfort (SMD 1.92 and 1.85) and psychological disability (SMD 1.66 and 1.87), both significantly higher than zero. The lowest was observed for functional limitation in Class II patients (SMD 0.78, 95% CI—0.11 to 1.67) and for physical disability in Class III (SMD 0.95, 95% CI 0.50 to 1.41). No test for subgroups was statistically significant, indicating that there were no differences between Class II and Class III. These results are consistent with meta-analyses of the differences between SMD in Class II and Class III (Figure 5), which also showed statistically insignificant differences of small magnitude: SMD of OHIP-14 scores ranged from 0.26 (95% CI—0.35 to 0.87; *I*^2^ = 68%) in functional limitation to 0.01 (95% CI −0.90 to 0.92; *I*^2^ = 85%) in physical disability. The SMD between Class II and Class III patients on the OQLQ and OHIP-14 total score was −0.03 (95% CI—0.61 to 0.54).

### 3.6. Sensivity Analysis

**Figure 6:**
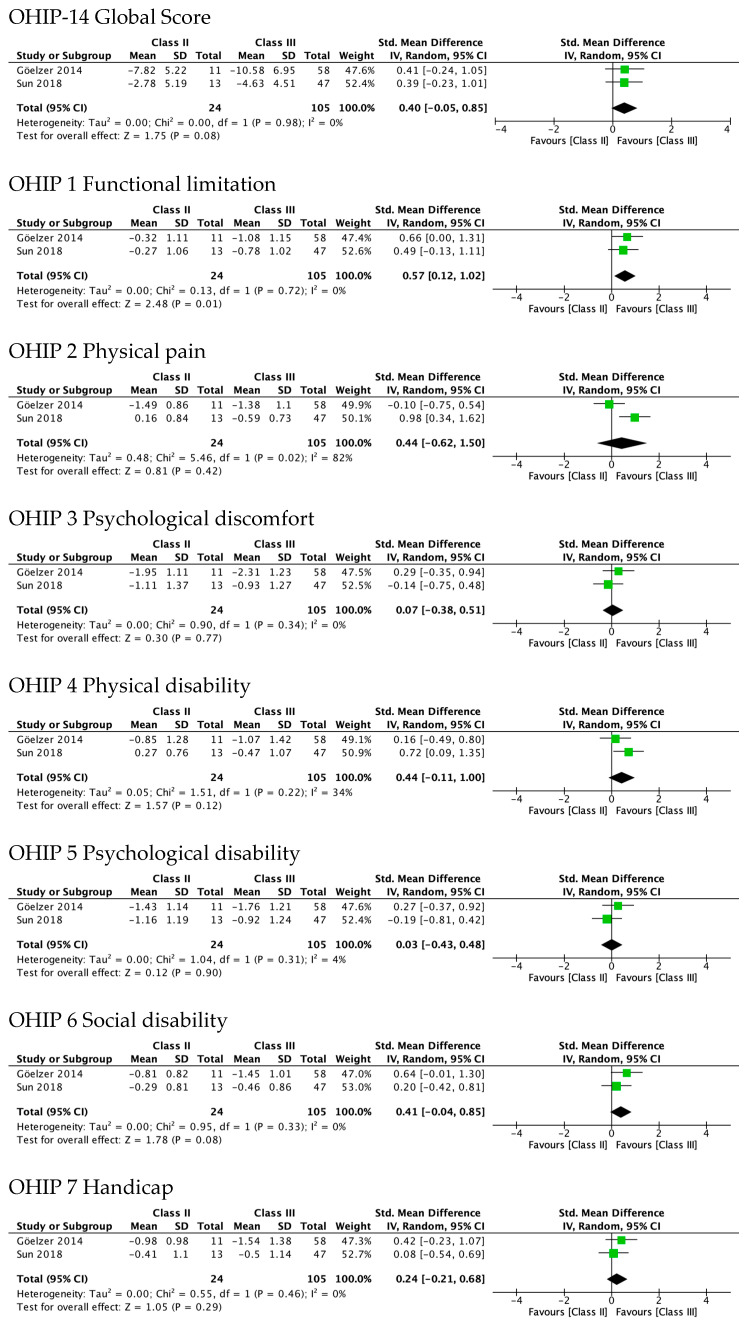
Results of sensitivity analysis after excluding studies with weak methodological quality. Meta-analysis of the differences between Class II and Class III standard mean differences (from pre-surgery to 4–7 months after surgery) in OHRQoL global scores and OHIP-14 domains’ scores.

Figure 6 shows sensitivity analyses performed after excluding the two studies rated as weak in their methodological quality [15,23]. The results from meta-analysis of the differences between Class II and Class III showed that it was only statistically significant in functional limitation (SMD 0.57, 95% CI 0.12–1.02). The difference in the domain of physical disability was of almost-moderate magnitude, but not statistically significant (SMD 0.44, 95% CI −0.11 to 1.00).

Consistently, the Supplementary Figure S1 shows that the test for subgroups between Class II and Class III was statistically significant in the domain of functional limitation (*p* = 0.02). In fact, Class II patients did not improve significantly (0.32, 95% CI—0.25 to 0.89; *I*^2^ = 0%), while those in Class III did (1.06, 95% CI 0.77 to 1.35; *I*^2^ = 0%). This pattern was also observed in the domain of physical disability (SMD 0.17 and 0.77) although the test for subgroups was not statistically significant.

### 3.7. Reporting Bias

Funnel plots to explore possible publication biases were not constructed, as we did not have more than 10 studies to pool in any meta-analysis.

### 3.8. Certainty of Evidence

All studies included were of observational design; therefore, the quality of evidence starts out low (Table 2). In addition, the quality of evidence was downgraded, mainly due to methodological limitations, inconsistency, and imprecision. Risk of bias was serious.

Due to concerns regarding blinding and withdrawals/dropouts, serious inconsistency downgraded certainty one level due to considerable heterogeneity and imprecision in one outcome. Indirectness of results was considered not serious since the studies included appropriately answer the question in terms of population, intervention, comparison, and results studied. Publication bias was also rated as not serious.

## 4. Discussion

### 4.1. Main Findings

The OHRQoL of patients with dentofacial deformities of Class II and III improved after orthognathic surgery. Improvement was of large magnitude in the global scores of both OHRQoL instruments applied in the studies, OQLQ and OHIP-14, and also in all OHIP-14 domains. No statistically significant differences by type of dentofacial deformity were found, but the sensitivity analyses (after excluding studies with weak methodological quality) showed that Class III patients’ improvement in the functional limitation domain was significantly higher than that of Class II patients. However, there was uncertainty in determining whether the type of dentofacial deformity affects the impact of orthognathic surgery on OHRQoL.

Findings obtained through the meta-analysis show improvement of large magnitude in all domains for both types of dentofacial deformities, but in Class II patients, only psychological domains (discomfort and disability), social disability, and handicap were statistically significant (not functional limitation, physical pain, and disability). Few studies incorporating Patient-Reported Outcomes (PROs) to measure the impact of orthognathic surgery on OHRQoL provided data for each type of dentofacial deformity, and sample sizes of Class II patients are smaller than those of Class III. Therefore, confidence intervals of the summary estimators obtained in our meta-analysis are very wide, especially for Class II patients.

On the other hand, the studies included in our meta-analysis used mostly the OHIP-14 [15,24,25], which was not specifically designed to measure the impact of orthognathic surgery. The OQLQ, specifically designed for this purpose, could have a higher sensitivity and show a greater improvement and a more precise estimator [40]. Therefore, studies using condition-specific instruments such as the OQLQ can allow one to distinguish the gradual impact of the treatment through the severity of the dentofacial deformity.

Previous systematic reviews consistently showed a positive impact of orthognathic surgery on OHRQoL in general [17,18,19,20,21,22], but none of them considered the type of dentofacial deformity. A meta-analysis of results focused on the OQLQ, specifically designed to measure the impact of the orthognathic surgery [20], showed improvement in the overall score and in the domains of social aspects and facial aesthetics. We also found improvement in the OQLQ global score, but we could not construct a meta-analysis by OQLQ domain since only one study [24] stratified according to type of dentofacial deformity reported data by domains. Statistically significative improvement for all domains of the OQLQ and OHIP-14 was estimated by two other systematic reviews with meta-analyses [21,22], which was consistent with our results in Class III patients. However, we found no statistically significant improvement in physical pain or physical disability of OHIP-14 in Class II patients.

In accordance with previous research [21], our findings showed a very low level of quality of evidence due to methodological limitations. Most studies included in our systematic review had an uncontrolled before–after design, which limited capacity to control for all relevant potential confounders due to lack of randomization and, therefore, they are more vulnerable to bias.

The sensitivity analysis performed to take into account the methodological quality of primary studies is especially relevant in this context. After excluding the methodologically weak quality studies, improvement differences between Class III and Class II patients were greater in the functional limitations, physical pain, and physical disability domains of OHIP-14. In these domains, improvement was of large magnitude and statistically significant in Class III patients, but small or moderate and not statistically significant in Class II patients. These findings support the need for further research to clarify the impact of orthognathic surgery considering the type of dentofacial deformities.

There is high variability among the studies regarding the time assessment, especially before surgery. Some studies considered the baseline as the stage prior to any intervention (before the installation of pre-surgical orthodontic appliances), other studies just before surgery, and a few in at undetermined timepoint of the pre-surgical period. Between the evaluation before pre-surgical orthodontic treatment and the preoperative phase, just before surgery, studies reported a significant worsening in OHRQoL [20,39,41] due to pre-surgical orthodontic decompensation.

Although the real impact of orthodontic–surgical treatment should be obtained by comparing the baseline measurement before the installation of pre-surgical orthodontic appliances with the end of the treatment, when the postsurgical orthodontics have been removed, only one study had this design [15]. This study showed statistically significant improvement (from before the installation of pre-surgical orthodontic appliances to after removal of the postsurgical orthodontics) in the OHIP-14 global score and all its domains in Class II and Class III patients [15]. This improvement was of a large magnitude except in functional limitation, physical pain, and physical disability in Class III patients. The other studies with OHRQoL assessment before the installation of pre-surgical orthodontic appliances [13,33,34,38] did not clarify whether the last follow-up occurred after removal of the postsurgical orthodontics.

The time point when the follow-up assessment was performed in the studies included in our meta-analysis ranges from 4 to 7 months after surgery. Changes in functional and facial aesthetics resulting from orthognathic surgery are dependent on the stability of surgical procedures [42]. The authors agree that the profile has attained its definitive configuration after 6 months [43] since edema and muscular readaptation are expected to resolve between 6 and 12 months [44,45]. Although there is a lack of consensus on the suitable times to assess the impact of orthognathic surgery, studies extending follow-up beyond 6 months are necessary to estimate the real impact of the orthognathic surgery.

### 4.2. Strengths and Limitations

The present study was strictly conducted in accordance within the guidelines of the Cochrane Handbook for the Systematic Review of Interventions [26]. Even though the impact of orthognathic surgery on OHRQoL is a subject of great interest and assessed in numerous studies, only a few of them analyze the differences according to the type of dentofacial deformities.

The variability among studies on the assessment times before and after surgery limited their inclusion in the meta-analysis. In addition, clinical diversity due to variability in the participants according to age, gender, or degree of severity of dentofacial deformity introduced considerable heterogeneity. However, in order to minimize this, we used a random-effects model in the analysis.

Finally, our findings should be interpreted with caution due to the very low certainty of the evidence, which translates into a significant uncertainty of the real magnitude of the impact of dentofacial deformities on the OHRQoL.

### 4.3. Implications for Practice and Research

The quantitative synthesis of results obtained in studies with moderate methodological quality suggests differences according to types of dentofacial deformities in the magnitude of the OHRQoL improvement experienced by patients.

Future research comparing types of dentofacial deformities, measuring OHRQoL with condition-specific instruments such as the OQLQ, and with robust methods are needed to clarify this issue. It is relevant to incorporate PROs as a measure of the patient’s perspective, and not only to evaluate the results of the treatment from the aesthetic and functional points of view. Sociocultural conditions and severity of the dentofacial deformity could influence the motivation for treatment and its impact on the OHRQoL.

## 5. Conclusions

There is not enough evidence to support differences between Class II and III patients in the OHRQoL impact at 4–7 months follow-up after orthognathic surgery. However, sensitive analyses excluding those studies with weak methodological quality suggest differences according to these types of dentofacial deformities in the domains of functional limitation, physical pain, and physical disability.

## Figures and Tables

**Table 1:** Characteristics of included studies and main results.

Study ID (Country)	Study Design (Instrument)	Sample Size	Age, Mean (SD) [Range] Gender	Pre- & Post-Surgical Assessment	Inclusion in Metanalyses or Reason for Exclusion	Changes after Surgery (during Pre-Surgical Stage *)
**Class I, II & III**						
Göelzer 2014 (Brazil)	Not Randomized No control group (OHIP-14)	Total = 74 Class I = 5 Class II = 11 Class III = 58	28 ys (9) (15–53) 66.2% women	Before surgery 4–6 months after surgery	Included	Class II: significant improvements in global score and all domains except functional limitation. Class III: significant improvements in global score and all domains.
Kurabe 2016 (Japan)	Not Randomized With control group (OHIP-J54)	Total = 65 Class I = 10 Class II = 8 Class III = 47	23.6 ys (8.1) (15–43) 67.7% women	Before surgery 6 months after surgery	Median and interquartile range, not reported mean (SD)	Class II: no statistically significant change. Class III: significant improvement.
Sun 2018 (China)	Not Randomized With control group (OHIP-14) (OQLQ)	Total = 85 Class I = 18 Class II = 15 Class III = 52	24 ys (17–41) 63.5% women	Before surgery 5–7 months after surgery	Included	Class II: significant improvement in discomfort and disability OHIP-14 domains; and social aspects, facial aesthetic, and global score of OQLQ. Class III: significant improvement in all scores of the OHIP-14 and OQLQ.
**Class II & III**						
Baherimoghaddam 2016 (Iran)	Not Randomized No control group (OHIP-14)	Total = 58 Class II = 28 Class III = 30	25.1 ys (3.4) CII; 21.3 ys (2.7) CIII 46.6% women	Before pre-surgical orthodontics Before surgery 6 months after surgery 12 months after debonding	Included	During pre-surgical stage *: Class II: significant worsening in psychological discomfort. Class III: significant worsening in psychological discomfort and psychological disability. Six months after surgery: Class II and III: significant improvements in all domains.
Eslamipour 2017 (Iran)	Not Randomized No control group (OQLQ)	Total = 43 Class II = 13 Class III = 30	Age not reported 69.8% women	The last visit (10–20 days) before surgery 3 weeks, 3 & 6 months after surgery	Included	Class II and III: significant improvement at 3 and 6 months after surgery in global score.
Tuk 2021 (Netherlands)	Not Randomized No control group (OHIP-14)	Total = 85 Class I = 1 Class II = 55 Class III = 29	28.6 ys (10.6) (18–60) 56.5% women	Before surgery Every day for the first 7 days, 4 weeks, 6 months, at least 1 year after surgery	Not reported: the n by class in follow-ups	Class II and III: significant worsening at 4 weeks, and significant improvement at 6 and 12 months after surgery.
**Only Class II**						
Bergamaschi 2021 (Brazil)	Not Randomized No control group (OHIP-14)	Total = 43	31 ys (18–66] 76.7% women	Before surgery (1 week) 6–12 months after surgery (mean follow-up 9 months)	Median (Min–Max) not reported mean (SD)	Significant improvement after surgery in overall OHIP-14 and all domains except functional limitation and physical disability.
**Only Class III**						
Rustemeyer 2012 (Germany)	Not Randomized No control group (OHIP-14)	Total = 30	24.3 ys (4.5) 56.7% women	Before pre-surgical orthodontics Mean 8.3 months after surgery	Baseline before any orthodontic treatment	Significant improvement in psychological discomfort and social disability.
Chaurasia 2018 (Nepal)	Not Randomized No control group (OHIP-14)(OQLQ)	Total = 14	21.78 ys (2.29) 35.7% women	Before surgery 8–12 months after surgery (mean 9.2)	Follow-up: mean 9.2 months	OHIP-14: significant improvement in global score and all domains except social disability and handicap. OQLQ: significant improvement in global score and all domains.
Tachiki 2018 (Japan)	Not Randomized No control group (OQLQ)	Total = 20	23.2 ys (7.3) 50% women	Before pre-surgical orthodontics At least 4 months after setting orthodontic appliances At least 3 months after surgery	Undetermined period before surgery	During the pre-surgical stage *: worsening in all domains. Three months after surgery: significant improvement in all domains, except awareness.
Geramy 2019 (Iran)	Not Randomized No control group (OHIP-14)	Total = 29	24.23 ys (4.2) 58.6% women	Before pre-surgical orthodontics 6 months after surgery	Baseline before any orthodontic treatment	Significant changes in items related to physical pain and physical disability (OH-4, OH-8) and psychological domain (OH-9, OH-10).
Ni 2019 (China)	Not Randomized With control group (OHIP-14)(OQLQ)	Total = 21	24.1 ys (3.67) (18–33] 47.6% women	Before pre-surgical orthodontics 6–8 months after setting orthodontic appliances 6–8 months after surgery	Undetermined period before surgery	(During the pre-surgical stage *: significant worsening in OQLQ domains, except for awareness and social aspects.) 6 to 8 months after surgery: significant improvement in all domains.
Chadda 2021 (India)	Not Randomized No control group (OHIP-14)	Total = 28	23.78 ys (1.36) (21–26] 57.1% women	Before surgery 6 months after surgery	Not reported scores, results reported by items	Significant improvement in all items, except for OH2, OH3, OH7, OH8m and OH14.

* During pre-surgical stage: between the evaluation before pre-surgical orthodontic treatment and the pre-operative phase, just before surgery.

**Table 2:** Summary of findings for the main results.

Certainty Assessment							№ of Patients		Effect		Certainty
№ of Studies	Study Design	Risk of Bias	Inconsistency	Indirectness	Imprecision	Other Considerations	Dentofacial	Comparison	Relative (95% CI)	Absolute (95% CI)	
Global score Class II (before–after surgery)											
4	observational studies	serious ^a^	serious ^b^	not serious	not serious	strong association	65	65	-	SMD 2.09 higher (0.68 higher to 3.49 higher)	⨁◯◯◯ VERY LOW
Global score Class III (before–after surgery)											
4	observational studies	serious ^a^	serious ^b^	not serious	not serious	strong association	165	165	-	SMD 1.96 higher (1.23 higher to 2.7 higher)	⨁◯◯◯ VERY LOW
Global score Class II vs. Class III											
4	observational studies	serious ^c^	serious ^d^	not serious	serious ^e^	none	65	165	-	SMD 0.03 lower (0.61 lower to 0.54 higher)	⨁◯◯◯ VERY LOW

**CI:** Confidence interval; **SMD:** Standardized mean difference. Explanations: ^a.^ We downgraded the evidence by one level because of serious concerns regarding risk of bias: Half of the studies have weak methodological quality. ^b.^ We downgraded the evidence by one level because of inconsistency: Considerable heterogeneity. ^c.^ We downgraded the evidence by one level because of serious concerns regarding risk of bias: Two studies have weak methodological quality. ^d.^ We downgraded the evidence by one level because of inconsistency: Substantial heterogeneity. ^e.^ We downgraded the evidence by one level because of serious concerns regarding imprecision: The studies include both appreciable benefit and appreciable harm.

## Data Availability

The data and materials supporting the conclusions of this manuscript are included in the article.

## Supplementary Materials

The following supporting information can be downloaded at: https://www.mdpi.com/article/10.3390/ijerph19041940/s1: Figure S1. Results of sensitivity analysis after excluding studies with weak methodological quality. Meta-analysis of the change from pre-surgery to 4–7 months after surgery in the OHIP-14 domain scores by type of dentofacial deformity; Table S1. Search strategy for each data base; Table S2. Checklist PRISMA 2020.

## Differences between the Protocol and the Review Conducted

The original title of the protocol was “Quality of life in patients with dentofacial deformities treated with orthognathic surgery. A systematic review”; it was changed to “Oral Health-related quality of life changes in patients with dentofacial deformities Class II and III after orthognathic surgery: a systematic review and meta-analysis” since we modified the main outcome. We also adjusted the age range of the included patients to those over 15 years old treated with conventional ortho-surgical technique.
