# Supplementary material for: Oral Health-Related Quality of Life Changes in Patients with Dentofacial Deformities Class II and III after Orthognathic Surgery: A Systematic Review and Meta-Analysis
Source: Int J Environ Res Public Health. 2022 Feb 9;19(4):1940. doi: 10.3390/ijerph19041940 (PMC8872566; doi:10.3390/ijerph19041940)

Supplementary Figure S1. Results of sensitivity analysis after excluding studies with weak methodological quality. Meta-analysis of the change from pre-surgery to 4 – 7 months after surgery in the OHIP-14 domain scores by type of dentofacial deformity.

OHIP-14 Global Score

Study or Subgroup	Std. Mean Difference	SE	Weight	Std. Mean Difference IV, Random, 95% CI
**3.8.1 Class II**				
Göelzer 2014	1.7409	0.5155	45.8%	1.74 [0.73, 2.75]
Sun 2018	0.6275	0.4034	54.2%	0.63 [−0.16, 1.42]
**Subtotal (95% CI)**			**100.0%**	**1.14 [0.05, 2.23]**
Heterogeneity: $Tau^2 = 0.41$; $Chi^2 = 2.89$, df = 1 (P = 0.09); $I^2 = 65\%$				
Test for overall effect: Z = 2.05 (P = 0.04)				
**3.8.2 Class III**				
Göelzer 2014	1.8607	0.2235	50.2%	1.86 [1.42, 2.30]
Sun 2018	1.2555	0.2265	49.8%	1.26 [0.81, 1.70]
**Subtotal (95% CI)**			**100.0%**	**1.56 [0.97, 2.15]**
Heterogeneity: $Tau^2 = 0.13$; $Chi^2 = 3.62$, df = 1 (P = 0.06); $I^2 = 72\%$				
Test for overall effect: Z = 5.15 (P < 0.00001)				
Test for subgroup differences: $Chi^2 = 0.44$, df = 1 (P = 0.51), $I^2 = 0\%$				

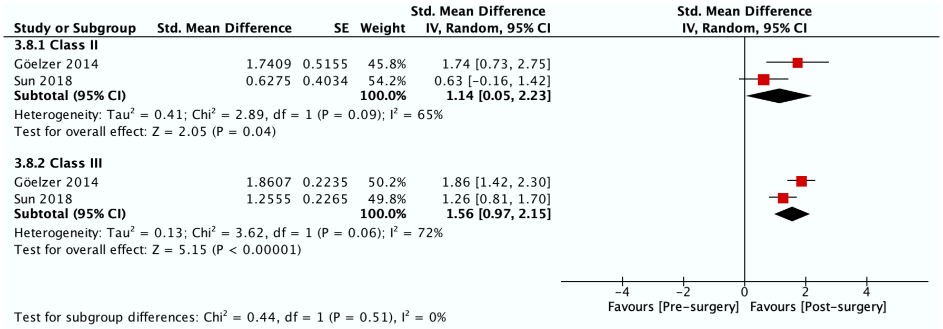

OHIP 1 Functional limitation

Study or Subgroup	Std. Mean Difference	SE	Weight	Std. Mean Difference IV, Random, 95% CI
**3.1.1 Class II**				
Göelzer 2014	0.3454	0.4303	45.7%	0.35 [−0.50, 1.19]
Sun 2018	0.3035	0.3949	54.3%	0.30 [−0.47, 1.08]
**Subtotal (95% CI)**			**100.0%**	**0.32 [−0.25, 0.89]**
Heterogeneity: $Tau^2 = 0.00$; $Chi^2 = 0.01$, df = 1 (P = 0.94); $I^2 = 0\%$				
Test for overall effect: Z = 1.11 (P = 0.27)				
**3.1.2 Class III**				
Göelzer 2014	1.1683	0.2014	53.9%	1.17 [0.77, 1.56]
Sun 2018	0.9391	0.2178	46.1%	0.94 [0.51, 1.37]
**Subtotal (95% CI)**			**100.0%**	**1.06 [0.77, 1.35]**
Heterogeneity: $Tau^2 = 0.00$; $Chi^2 = 0.60$, df = 1 (P = 0.44); $I^2 = 0\%$				
Test for overall effect: Z = 7.19 (P < 0.00001)				
Test for subgroup differences: $Chi^2 = 5.14$, df = 1 (P = 0.02), $I^2 = 80.5\%$				

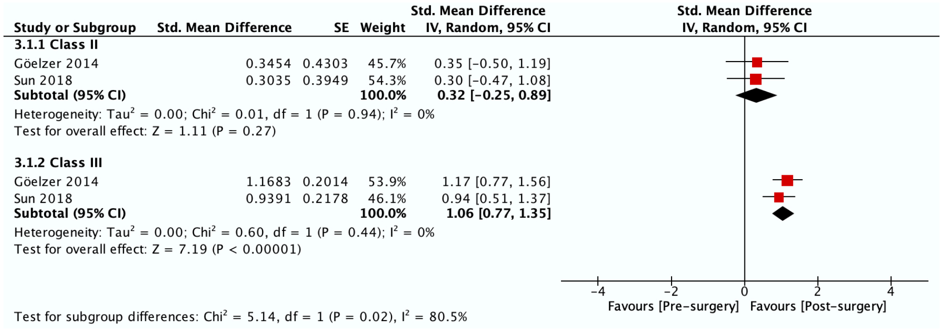

OHIP 2 Physical pain

Study or Subgroup	Std. Mean Difference	SE	Weight	Std. Mean Difference IV, Random, 95% CI
**3.2.1 Class II**				
Göelzer 2014	1.7296	0.5144	48.5%	1.73 [0.72, 2.74]
Sun 2018	−0.1886	0.3933	51.5%	−0.19 [−0.96, 0.58]
**Subtotal (95% CI)**			**100.0%**	**0.74 [−1.14, 2.62]**
Heterogeneity: $Tau^2 = 1.63$; $Chi^2 = 8.78$, df = 1 (P = 0.003); $I^2 = 89\%$				
Test for overall effect: Z = 0.77 (P = 0.44)				
**3.2.2 Class III**				
Göelzer 2014	1.3138	0.2054	50.8%	1.31 [0.91, 1.72]
Sun 2018	0.8168	0.2151	49.2%	0.82 [0.40, 1.24]
**Subtotal (95% CI)**			**100.0%**	**1.07 [0.58, 1.56]**
Heterogeneity: $Tau^2 = 0.08$; $Chi^2 = 2.79$, df = 1 (P = 0.09); $I^2 = 64\%$				
Test for overall effect: Z = 4.30 (P < 0.0001)				
Test for subgroup differences: $Chi^2 = 0.11$, df = 1 (P = 0.74), $I^2 = 0\%$				

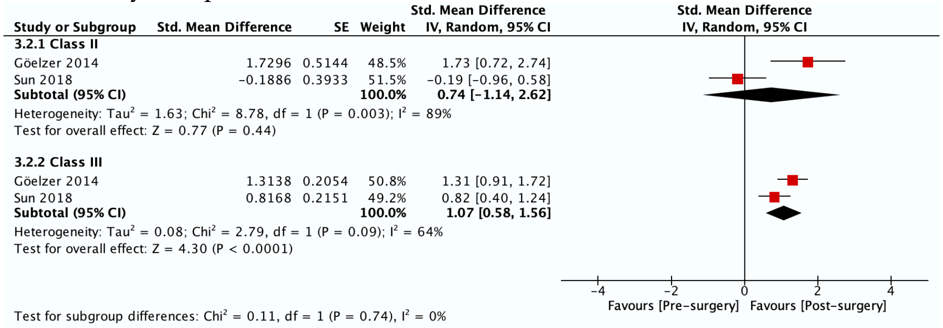

OHIP 3 Psychological discomfort

Study or Subgroup	Std. Mean Difference	SE	Weight	Std. Mean Difference IV, Random, 95% CI
**3.3.1 Class II**				
Göelzer 2014	2.1712	0.5589	45.0%	2.17 [1.08, 3.27]
Sun 2018	1.0129	0.4208	55.0%	1.01 [0.19, 1.84]
**Subtotal (95% CI)**			**100.0%**	**1.53 [0.40, 2.66]**
Heterogeneity: $Tau^2 = 0.43$; $Chi^2 = 2.74$, df = 1 (P = 0.10); $I^2 = 64\%$				
Test for overall effect: Z = 2.66 (P = 0.008)				
**3.3.2 Class III**				
Göelzer 2014	2.4044	0.2455	49.7%	2.40 [1.92, 2.89]
Sun 2018	0.933	0.2177	50.3%	0.93 [0.51, 1.36]
**Subtotal (95% CI)**			**100.0%**	**1.66 [0.22, 3.11]**
Heterogeneity: $Tau^2 = 1.03$; $Chi^2 = 20.11$, df = 1 (P < 0.00001); $I^2 = 95\%$				
Test for overall effect: Z = 2.26 (P = 0.02)				
Test for subgroup differences: $Chi^2 = 0.02$, df = 1 (P = 0.89), $I^2 = 0\%$				

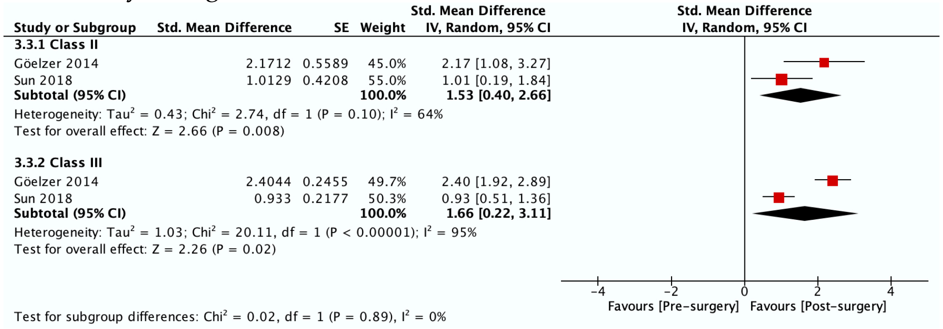

OHIP 4 Physical disability

Study or Subgroup	Std. Mean Difference	SE	Weight	Std. Mean Difference IV, Random, 95% CI
**3.4.1 Class II**				
Göelzer 2014	0.809	0.4471	48.7%	0.81 [−0.07, 1.69]
Sun 2018	−0.4409	0.3978	51.3%	−0.44 [−1.22, 0.34]
**Subtotal (95% CI)**			**100.0%**	**0.17 [−1.06, 1.39]**
Heterogeneity: $Tau^2 = 0.60$; $Chi^2 = 4.36$, df = 1 (P = 0.04); $I^2 = 77\%$				
Test for overall effect: Z = 0.27 (P = 0.79)				
**3.4.2 Class III**				
Göelzer 2014	0.9608	0.1965	51.7%	0.96 [0.58, 1.35]
Sun 2018	0.5565	0.2104	48.3%	0.56 [0.14, 0.97]
**Subtotal (95% CI)**			**100.0%**	**0.77 [0.37, 1.16]**
Heterogeneity: $Tau^2 = 0.04$; $Chi^2 = 1.97$, df = 1 (P = 0.16); $I^2 = 49\%$				
Test for overall effect: Z = 3.79 (P = 0.0002)				
Test for subgroup differences: $Chi^2 = 0.83$, df = 1 (P = 0.36), $I^2 = 0\%$				

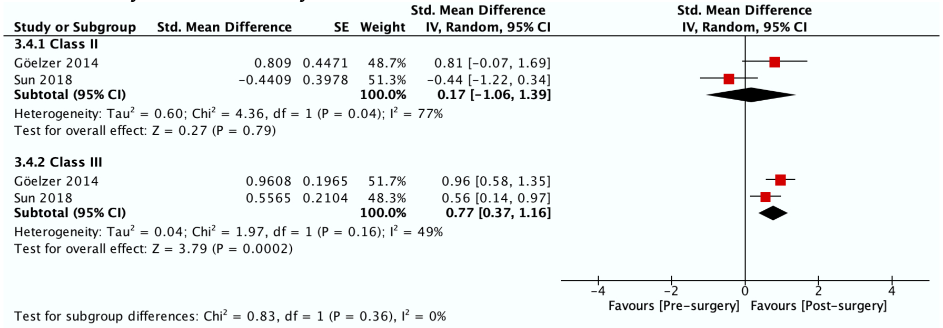

OHIP 5 Psychological disability

Study or Subgroup	Std. Mean Difference	SE	Weight	Std. Mean Difference IV, Random, 95% CI
**3.5.1 Class II**				
Göelzer 2014	1.6132	0.5038	42.8%	1.61 [0.63, 2.60]
Sun 2018	1.2667	0.4361	57.2%	1.27 [0.41, 2.12]
**Subtotal (95% CI)**			**100.0%**	**1.42 [0.77, 2.06]**
Heterogeneity: $Tau^2 = 0.00$; $Chi^2 = 0.27$, df = 1 (P = 0.60); $I^2 = 0\%$				
Test for overall effect: Z = 4.29 (P < 0.0001)				
**3.5.2 Class III**				
Göelzer 2014	1.9335	0.2262	49.8%	1.93 [1.49, 2.38]
Sun 2018	0.9856	0.219	50.2%	0.99 [0.56, 1.41]
**Subtotal (95% CI)**			**100.0%**	**1.46 [0.53, 2.39]**
Heterogeneity: $Tau^2 = 0.40$; $Chi^2 = 9.06$, df = 1 (P = 0.003); $I^2 = 89\%$				
Test for overall effect: Z = 3.08 (P = 0.002)				
Test for subgroup differences: $Chi^2 = 0.01$, df = 1 (P = 0.94), $I^2 = 0\%$				

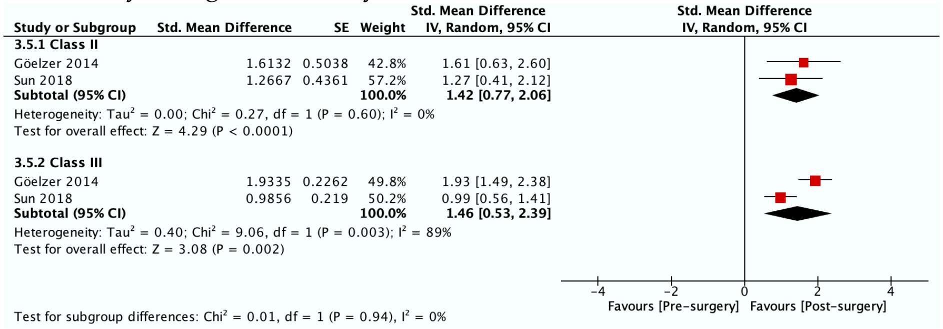

OHIP 6 Social disability

Study or Subgroup	Std. Mean Difference	SE	Weight	Std. Mean Difference IV, Random, 95% CI
**3.6.1 Class II**				
Göelzer 2014	1.0059	0.4581	43.6%	1.01 [0.11, 1.90]
Sun 2018	0.3615	0.396	56.4%	0.36 [−0.41, 1.14]
**Subtotal (95% CI)**			**100.0%**	**0.64 [0.02, 1.27]**
Heterogeneity: $Tau^2 = 0.02$; $Chi^2 = 1.13$, df = 1 (P = 0.29); $I^2 = 12\%$				
Test for overall effect: Z = 2.01 (P = 0.04)				
**3.6.2 Class III**				
Göelzer 2014	1.5983	0.2142	49.9%	1.60 [1.18, 2.02]
Sun 2018	0.5718	0.2106	50.1%	0.57 [0.16, 0.98]
**Subtotal (95% CI)**			**100.0%**	**1.08 [0.08, 2.09]**
Heterogeneity: $Tau^2 = 0.48$; $Chi^2 = 11.68$, df = 1 (P = 0.0006); $I^2 = 91\%$				
Test for overall effect: Z = 2.11 (P = 0.03)				
Test for subgroup differences: $Chi^2 = 0.53$, df = 1 (P = 0.46), $I^2 = 0\%$				

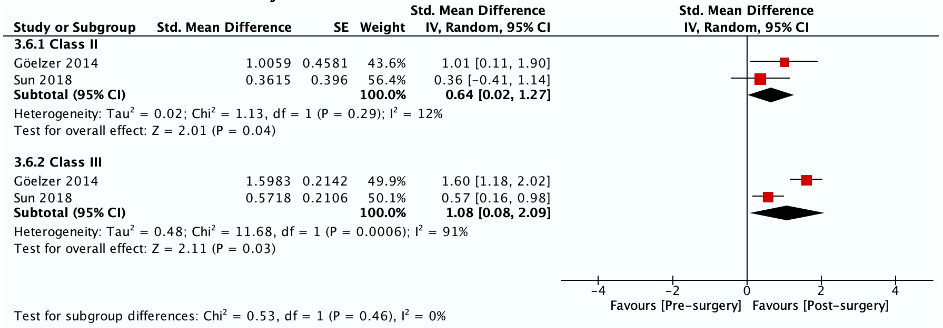

OHIP 7 Handicap

Study or Subgroup	Std. Mean Difference	SE	Weight	Std. Mean Difference IV, Random, 95% CI
**3.7.1 Class II**				
Göelzer 2014	1.2207	0.4723	44.3%	1.22 [0.30, 2.15]
Sun 2018	0.4677	0.3985	55.7%	0.47 [−0.31, 1.25]
**Subtotal (95% CI)**			**100.0%**	**0.80 [0.07, 1.53]**
Heterogeneity: $Tau^2 = 0.09$; $Chi^2 = 1.48$, df = 1 (P = 0.22); $I^2 = 33\%$				
Test for overall effect: Z = 2.14 (P = 0.03)				
**3.7.2 Class III**				
Göelzer 2014	1.435	0.209	50.0%	1.44 [1.03, 1.84]
Sun 2018	0.5718	0.2106	50.0%	0.57 [0.16, 0.98]
**Subtotal (95% CI)**			**100.0%**	**1.00 [0.16, 1.85]**
Heterogeneity: $Tau^2 = 0.33$; $Chi^2 = 8.46$, df = 1 (P = 0.004); $I^2 = 88\%$				
Test for overall effect: Z = 2.33 (P = 0.02)				
Test for subgroup differences: $Chi^2 = 0.13$, df = 1 (P = 0.72), $I^2 = 0\%$				

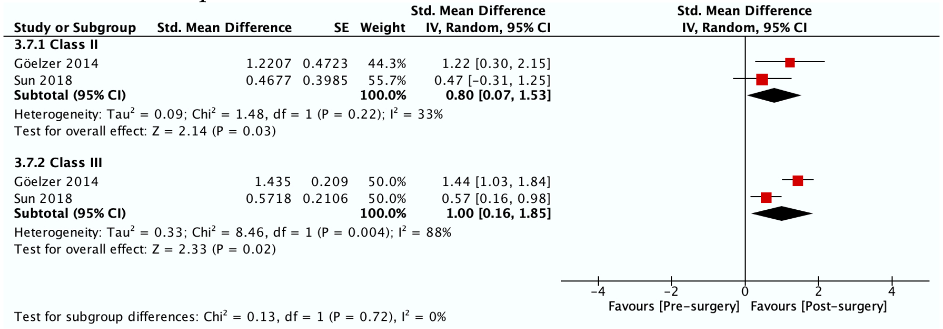

Supplement: Supplementary file 1 [file ijerph-19-01940-s001.zip › Supplementary Figure S1.pdf]
