# Supplementary material for: Oral Health-Related Quality of Life Changes in Patients with Dentofacial Deformities Class II and III after Orthognathic Surgery: A Systematic Review and Meta-Analysis
Source: Int J Environ Res Public Health. 2022 Feb 9;19(4):1940. doi: 10.3390/ijerph19041940 (PMC8872566; doi:10.3390/ijerph19041940)
Supplement: Supplementary file 1 [file ijerph-19-01940-s001.zip › Supplementary Table S1.pdf]

Table S1. Search Strategy for each Data Base.

Data Base	Search Strategy
PubMed	(((((orthognathic[Title/Abstract]) OR (dentofacial deformities[Title/Abstract])) OR (orthognathic surgery[Title/Abstract])) OR (dentofacial deformity[Title/Abstract])) OR (orthognathic surgery[MeSH Terms])) AND (((quality of life[Title/Abstract]) OR ("health related quality of life"[Title/Abstract])) OR (quality of life[MeSH Terms]))
Embase	('quality of life' OR 'health related quality of life questionnaire') AND ('dentofacial deformity'/exp OR 'orthognathic surgery')
Cochrane Central Register of Controlled Trials (CENTRAL)	((dentofacial deformity):ti,ab,kw OR (orthognathic surgery):ti,ab,kw) AND ((quality of life) OR (health related quality f life):ti,ab,kw OR (oral health related quality of life):ti,ab,kw)
Scopus	((TITLE-ABS-KEY (dentofacial AND deformity) OR TITLE-ABS-KEY (dentofacial AND deformities))) OR (TITLE-ABS-KEY (orthognathic AND surgery)) AND ((TITLE-ABS-KEY (quality AND of AND life) OR TITLE-ABS-KEY (health AND related AND quality AND of AND life)))
